# The Effect of Calcium Supplementation on Body Weight Before and During Pregnancy in Women Enrolled in the WHO Calcium and Preeclampsia Trial

**DOI:** 10.1177/0379572120944671

**Published:** 2020-11-17

**Authors:** Gabriela Cormick, Ana Pilar Betrán, Janetta Harbron, Armando Seuc, Cintia White, James M. Roberts, Jose M. Belizán, G. Justus Hofmeyr

**Affiliations:** 1 Department of Mother and Child Health Research, 172472 Institute for Clinical Effectiveness and Health Policy (IECS-CONICET), Buenos Aires, Argentina; 2 Department of Human Biology, Faculty of Health Sciences, 37716 University of Cape Town, South Africa; 3 Departamento de Salud, 28224 Universidad Nacional de La Matanza, San Justo, Argentina; 4 HRP–UNDP/UNFPA/UNICEF/WHO/World Bank Special Programme of Research, Development and Research Training in Human Reproduction, Department of Reproductive Health and Research, 3489 World Health Organization, Geneva, Switzerland; 5 217256 Instituto Nacional de Higiene, Epidemiología y Microbiología, La Habana, Cuba; 6 Magee-Womens Research Institute, Department of Obstetrics and Gynecology, Epidemiology and Clinical and Translational Research 6614 University of Pittsburgh, PA, USA; 7 Effective Care Research Unit, 37707 Universities of the Witwatersrand, Walter Sisulu and Fort Hare, South Africa; 8 University of Botswana, Gaborone, Botswana

**Keywords:** obesity, calcium, pregnancy, supplementation, body mass index

## Abstract

**Introduction:**

Obesity is a major and challenging public health problem. The aim of this substudy is to evaluate the effect of calcium supplementation on body weight in women recruited in the Calcium and Preeclampsia trial.

**Methods:**

Women were recruited before pregnancy and randomized to receive a calcium supplement containing 500 mg of elemental calcium or placebo until 20 weeks’ gestation; all women received 1.5 g from 20 weeks until delivery.

**Results:**

A total of 630 women conceived during the study, 322 allocated to calcium and 308 to placebo. Among these, 230 allocated to calcium and 227 allocated to placebo had information on body weight at baseline and at 8 weeks' gestation. During the study period, women allocated to calcium had a mean weight increase of 1.1 (SD ±5.5) kg, whereas those allocated to placebo had a mean increase of 1.5 (SD ±6.1) kg, a mean difference of 0.4 kg (95% −0.4 (−1.4 to 0.6); *P* = .408). Women classified as obese at the start of the trial had a lower body weight gain at 8 weeks’ gestation (1.0 kg; 95% CI: −3.2 to 1.2; *P* = .330) and at 32 weeks’ gestation (2.1 kg; 95% CI: 5.6-1.3; *P* = .225) if they received calcium as compared to placebo. However, none of these differences were statistically significant.

**Conclusion:**

The smaller increase in body weight found in women supplemented with 500 mg elemental calcium daily is quantitatively consistent with previous studies. However, in this study, the difference was not statistically significant.

## Introduction

Obesity represents a major and challenging public health problem as it increases the risk of developing high blood pressure, insulin resistance, heart disease, diabetes, osteoarthritis, and sleep apnoea.^1,2^ During pregnancy, obesity increases the risk of adverse pregnancy outcomes.^1,3^ Obesity before and during pregnancy increases the risks of gestational diabetes and hypertension, mental ill health before and after pregnancy, cesarean section, preterm birth, large for gestational age, miscarriage, stillbirth, and fetal death.^4^ It has been estimated that a higher 5 to 7 kg/m2 body mass prepregnancy index (BMI) doubles the risk of preeclampsia.^5,6^ Even an increase of 1 or 2 BMI units between pregnancies increases the risk of hypertension and gestational diabetes.^7^

Several studies have investigated the modulating effect of calcium intake upon body weight.^8^ In animal models, changes in lipid metabolism with calcium supplementation have been shown.^9,10^ In addition, after induced weight loss, obese mice on a diet low in calcium experienced increased lipogenesis and rapid bodyweight regain, whereas calcium supplementation inhibited weight regain.^11^

In order to improve pregnancy outcomes, overweight and obese women are advised to lose weight before conception; however, the evidence on how to achieve this is scarce.^7^ Also, for overweight or obese pregnant women, there is little evidence on how to manage their weight during pregnancy.^7,12^ Until more robust evidence is available, recommendations are to gain weight, although at a reduced rate, as dieting during pregnancy may increase the risk of ketosis that is harmful to the fetus. The US Institute of Medicine Guidelines recommend that women who are overweight should aim to gain 7 to 11.5 kg, and women who are obese should aim to gain 5 to 9 kg during the entire pregnancy.^13^

The Calcium and Preeclampsia (CAP) trial was a randomized controlled trial (RCT) conducted between 2011 and 2017 which aimed to test the effect of calcium supplementation commencing before pregnancy and continued up to 20 weeks’ gestation on the incidence of preeclampsia in women with a history of preeclampsia.14 Nested in the CAP trial, the aim of this substudy was to evaluate the effect of calcium supplementation on body weight in women at 8, 20 and 32 weeks’ gestation recruited in this trial.

## Methods

The protocol of the multicenter CAP trial has been previously published.^14^ The trial included women at high risk of preeclampsia from South Africa, Zimbabwe, and Argentina. Briefly, women were recruited before pregnancy and randomized to receive until 20 weeks’ gestation a calcium supplement containing 500 mg of elemental calcium as calcium carbonate or placebo identical in shape, color, and taste to the calcium tablet. Women were asked to chew the tablet during the day, not close in time to taking food or iron supplements. Following World Health Organization’s (WHO) recommendations, from 20 weeks’ gestation until delivery, all women received calcium supplementation (1.5 g).^15^ The trial recruited participants from July 12, 2011, to September 8, 2016. For the substudy presented here, our sample included all participants of the CAP trial with information on body weight registered at baseline and at 8 weeks of pregnancy.

### Sample Size

The CAP trial recruited 540 women with a pregnancy of more than 20 weeks.^16^ Of those, 457 women had information on body weight at 8 weeks’ gestation and were used for the analysis of this substudy.

### Measurements

Data collected at recruitment and used for the analysis of this substudy included maternal age, height and weight, country of residence, number of previous pregnancies, and date of birth of the last pregnancy complicated with preeclampsia. For women who became pregnant, weight during pregnancy was recorded at 8, 20, and 32 weeks’ gestation during the scheduled trial follow-up visits. Compliance to the study supplements was measured at each follow-up visit. Participants were asked to bring the study bottle to each visit and the remaining supplements were recorded. In those cases where the woman did not bring the bottle, they were phoned to assess the number of supplements taken. Compliance was calculated as a percentage of the number of used supplements of the total number of supplements that should have been taken from the start of the trial until 20 weeks’ gestation.

Research nurses specially trained for the CAP trial assessed all anthropometric and clinical measurement at admission (when women joined the study, were randomized, and started calcium and placebo supplementation) and during follow-up visits at each participating site.

Bodyweight was measured to the nearest 0.1 kg. Women were measured in light clothing and without shoes. Height was measured to the nearest 1 cm using a stadiometer while the participant was not wearing shoes. Scales and stadiometers were those provided by each hospital and remained the same throughout the study. The Manual of Operations and the Standard Operating Procedures provided instructions on how women should be weighed and measured.

Prepregnancy BMI was calculated as weight (kg) divided by the square of the body height (m) using measurements recorded at admission. Women were classified according to the WHO BMI standards for adults, defined as underweight (BMI < 18.5), normal (18.5 ≤ BMI < 25), overweight (BMI ≥ 25 and <30), or obese (BMI ≥ 30).^17^

Dietary energy and calcium intakes were measured at 20 weeks’ gestation using a triple-pass 24-hour recall adapted from the method developed by Nelson et al.^18^ Before the first CAP trial interview, it was pilot tested in Argentina, South Africa, and Zimbabwe.^19^

### Analysis

Baseline data were compared between the calcium and placebo groups for the population included in this subanalysis (women who were randomized at admission, became pregnant, and had weight measurements at 8 weeks’ gestation). We used an intention-to-treat approach for this analysis as recommended by the Consolidated Standards of Reporting Trials (CONSORT) guidelines.^20^

Continuous variables were described with means and SDs and categorical variables with percentages. To evaluate the effect of calcium on body weight, we analyzed the information collected from randomization to 8 weeks’ gestation as we did not have further weight measurements after randomization and before pregnancy. We used weight at 8 weeks’ gestation as a surrogate of preconceptual body weight. Evidence from a published cross-sectional study of 1000 women shows there is no significant change in mean maternal body weight or BMI in the first trimester.^21^

We evaluated the effect of calcium supplementation on body weight at 20 weeks’ gestation as the difference between weight at admission to the study and weight at 20 weeks’ gestation as all women began a similar calcium intake at 20 weeks’ gestation until the end of pregnancy. We also evaluated any difference at 32 weeks’ gestation. To compare continuous variables between the intervention and control groups, a *t* test was used, and to compare categorical values, a χ^2^ test was used.

We then performed the same analysis of the effect of calcium supplementation at 8, 20, and 32 weeks’ gestation for the following 3 BMI classification groups: underweight or normal, overweight, and obese. To explore the effect in compliant women, a subgroup analysis was conducted for women with compliance 80% or higher from admission to the corresponding follow-up visit.

Finally, as the time between admission and pregnancy was different for each woman, we evaluated the effect of calcium supplementation from admission to 8 weeks’ gestation according to the number of months of supplementation. On the basis of the literature, we proposed 3 groups in terms of the length of the calcium supplementation: (1) from 2 to less than 6 months, (2) from 6 to less than 12 months, and (3) more than 12 months, as there are studies showing effect with a duration between 2 and 6 months; however, there are no studies with a duration of less than 2 months and very few of more than 6 months. A *P* value of .05 was used to define significant differences. Statistical analysis was performed with SPSS version 21.

## Results

**Figure 1.**
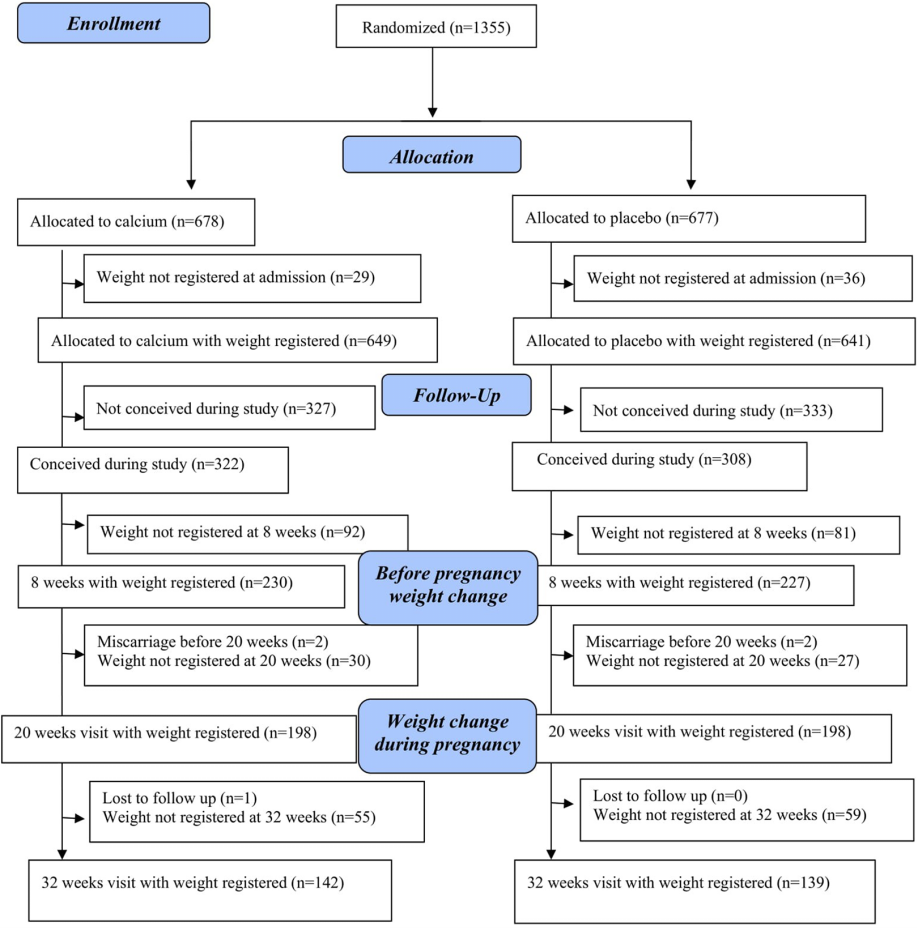
Women for the analysis of the effect of calcium on weight.

A total of 1355 women participated in the CAP study and were randomized to receive calcium (n = 678) or placebo (n = 677; see Figure 1). Of those, 630 women conceived during the study, with 322 in the calcium group and 308 in the placebo. Of these, 230 allocated to calcium and 227 allocated to placebo had information on body weight at 8 weeks’ gestation, 198 allocated to calcium and 198 allocated to placebo had information on body weight at 20 weeks’ gestation, and 142 allocated to calcium and 139 allocated to placebo had information on body weight at 32 weeks’ gestation. None of the participants required to interrupt calcium or placebo supplementation due to adverse events.

Baseline characteristics of all randomized women and women included in this substudy are illustrated in Table 1. We did not find any relevant difference at baseline in any of the variables between those allocated to calcium and placebo in any of the groups nor any difference between groups. The average duration of calcium or placebo supplementation from recruitment at baseline to 8 weeks’ gestation was 11.3 (SD ±8.8) months and 11.2 (SD ±9.3) months for those allocated to the calcium and placebo groups, respectively.

**Table 1.** Comparison of Baseline Characteristics of Women Included in the CAP Trial Study and Women Included in This Study.^a^

	Placebo			Calcium			Placebo			Calcium		
	N	Mean	SD	N	Mean	SD	N	Mean	SD	N	Mean	SD
Maternal age	677	30.4	5.9	678	30.2	5.8	227	29.0	5.0	230	29.7	5.4
Parity	677	2.0	1.1	678	1.9	1.1	227	1.9	1.1	230	2.0	1.1
Weight at ADM (kg)	641	76.9	18.8	649	76.2	18.5	227	74.0	16.3	230	75.8	16.8
Height at ADM (cm)	613	160.0	6.7	617	160.2	6.3	215	159.9	6.4	221	160.7	6.4
BMI at ADM	608	30.1	6.9	610	29.7	7.1	215	28.9	6.2	221	29.3	6.6
Months since last birth with PE	648	24.6	36.3	645	22.1	29.7	212	20.2	26.2	224	20.9	27.3
Time in study from ADM to 8 weeks’ gestation	247	11.15	9.07	247	11.16	8.68	227	11.25	9.28	230	11.26	8.79
Country of origin												
Argentina	57	(8.4%)		60	(8.8%)		8	(3.5%)		11	4.8	
South Africa	479	(70.8%)		477	(70.4%)		150	(66.1%)		147	63.9	
Zimbabwe	141	(20.8%)		141	(20.8%)		69	(30.4%)		72	31.3	

Abbreviations: ADM, admission; BMI, body mass prepregnancy index; CAP, Calcium And Preeclampsia; PE, preeclampsia.

^a^Mean values with SD.

Dietary calcium intake was only estimated using a triple-pass 24-hour recall at 20 weeks’ gestation. There was no difference in dietary calcium intake at 20 weeks’ gestation between the groups, those allocated to calcium (n = 143) had a mean dietary calcium intake of 418.9 (SD = 249.2) mg, whereas those allocated to placebo (n = 153) had a mean dietary calcium intake of 435.7 (SD = 348.9) mg.^22^

Women allocated to calcium had a weight mean increase of 1.1 (SD ±5.5) kg, whereas women allocated to placebo had a mean increase of 1.5 (SD ±6.1) kg, a mean difference of 0.4 kg (95% −0.4 (−1.4 to 0.6); *P* = .408; Table 2). From admission to 20 weeks, women allocated to calcium had an increase in weight of 3.9 kg (SD ±6.0) and those allocated to placebo 4.0 kg (SD ±7.0), a mean difference of 0.1 kg (95% −0.1 (−1.3 to 1.1); *P* = .811; Table 2). From admission to 32 weeks’ gestation, the weight of women allocated to calcium increased 7.7 kg (SD ±6.6) and those allocated to placebo 8.3 kg (SD ±7.3), a mean difference of 0.6 kg (95% −0.6 (−2.2 to 1.0); *P* = .457).

**Table 2.** Weight Change Between Admission and 8, 20, and 32 Weeks’ Gestation by Baseline BMI at Admission.

** **	Placebo		Calcium			*P* value^a^
	n	Mean difference (SD)	n	Mean difference (SD)	Mean difference (95% CI)	
Weight change at 8 weeks’ gestation	227	1.5 (6.1)	230	1.1 (5.5)	−0.4 (−1.4 to 0.6)	.408
BMI at ADM <25 kg/m^2^	72	0.8 (4.2)	70	1.5 (4.4)	0.7 (−0.8 to 2.1)	.334
25 ≤ BMI at ADM < 30 kg/m^2^	72	1.7 (5.3)	65	0.8 (4.0)	−0.9 (−2.5 to 0.7)	.261
BMI at ADM ≥ 30 kg/m^2^	83	2.0 (7.9)	95	0.9 (7.0)	−1.0 (−3.2 to 1.2)	.330
Weight change at 20 weeks’ gestation	198	4.0 (7.0)	198	3.9 (6.0)	−0.1 (−1.3 to 1.1)	.811
BMI at ADM <25 kg/m^2^	62	4.6 (5.7)	60	4.5 4.7)	−0.1 (−1.9 to 1.8)	.916
25 ≤ BMI at ADM < 30 kg/m^2^	63	3.9 (6.6)	53	3.4 (4.4)	−0.5 (−2.5 to 1.6)	.628
BMI at ADM ≥ 30 kg/m^2^	73	3.7 (8.2)	85	3.7 (7.6)	0.0 (−2.5 to 2.5)	1.000
Weight change at 32 weeks’ gestation	139	8.3 (7.3)	142	7.7 (6.6)	−0.6 (−2.2 to 1.0)	.457
BMI at ADM <25 kg/m^2^	44	8.2 (5.4)	42	9.2 (5.5)	1.1 (−1.3 to 3.4)	.398
25 ≤ BMI at ADM < 30 kg/m^2^	50	8.2 (6.6)	37	8.1 (5.0)	−0.1 (−2.6 to 2.4)	.936
BMI at ADM ≥ 30 kg/m^2^	45	8.6 (9.4)	63	6.5 (7.9)	−2.1 (−5.6 to 1.3)	.225

Abbreviations: ADM, admission; BMI, body mass prepregnancy index.

^a^ Differences were tested using a *t* test and a *P* value of .05.

Table 2 shows changes in body weight from admission to 8, 20, and 32 weeks’ gestation stratified by baseline BMI categories. Women who started the trial with BMI equal or higher than 30 had a lower body weight gain at 8 weeks’ gestation (1.0 kg; 95% −1.0 (−3.2 to 1.2); *P* = .330) and at 32 weeks’ gestation (2.1 kg; 95% −2.1 (−5.6 to 1.3); *P* = .225) if they received calcium as compared to placebo; however, none of these differences were statistically significant.

Supplementary Table S1 shows compliance of women measured at 8, 20, and 32 weeks’ gestation. We did not find any difference in compliance between calcium and placebo groups at any point. Between 50% and 60% of women had a compliance of 80% or more with the study supplements. When considering only participants with a compliance of 80% or more, we also found no statistically significant differences between the groups either (Supplementary Table S2). Finally, we analyzed the effect of calcium supplementation on body weight by time in the study between admission to pregnancy, and we found no difference between the calcium and placebo groups (Supplementary Table S3).

## Discussion

This study shows that supplementation with calcium prior to and during early pregnancy was associated with a small but consistent reduction in weight gain in comparison with women receiving a placebo. Although the results did not achieve our chosen level of statistical significance, mean values were consistently lower in the calcium supplementation groups. There was a greater effect in subgroups of obese and overweight women. Although this small effect may not seem clinically relevant, at a population level where a weight gain at midlife was estimated in 0.27 kg/yr, this effect could be important.^23,24^ Moreover, the tendency is consistent with observations in animal and human supplementation studies.^9,10,25^ Also, basic studies have described the mechanism that can explain the effect of calcium intake on body weight.^26^

Three mechanisms by which calcium could affect body weight have been postulated. Low calcium intake stimulates parathyroid hormone and 1-25 dihydroxyvitamin D secretion to increase calcium resorption from the bones, reabsorption from the kidneys, and absorption in the intestine in order to maintain specific calcium concentrations in extracellular fluids.^27,28^ However, higher levels of parathyroid hormone and 1-25 dihydroxyvitamin D also stimulate calcium influx into different cell types, including the adipocyte where it stimulates fatty acid synthase and lipogenesis.^27,28^ Low calcium diets have also been linked to insulin resistance and high blood pressure through similar collateral effects.^26,29-31^ In this way, hormones released to compensate for low serum calcium levels could produce an increase in blood pressure and lipogenesis.^29^ A second postulated mechanism is related to appetite regulation. Higher calcium intakes have been linked to increase in glucagon-like peptide 1 that reduces appetite.^32^ Finally, the third mechanism is associated with the fact that calcium absorption is inefficient; thus, a large proportion of calcium remains in the intestine where it can bind to bile acids or to fatty acids impairing their absorption and decreasing available energy.^33-39^

A systematic review including 6 studies from the United States and 1 from Iran with a total of 794 women showed that in obese adults, mainly nonpregnant women, calcium supplementation from 6 to 12 months compared to placebo had a greater effect to reduce body weight (0.74 kg; 95% CI: 1.00-0.48). These results are similar to the weight difference found in the obese subgroup of our study.^25^ However, the doses used in those studies were much higher than in our study. Six of the included studies had a duration of 6 months with a dose of 1000 mg of elemental calcium per day and 1 a duration of 24 months with a dose of 1500 mg of elemental calcium per day. The lower dose in our study may explain the lack of significance.

The results of this study should foster research on the role of calcium intake on body weight including basic and clinical studies to assess if achieving an adequate calcium intake can contribute to long-term weight reduction. In addition, adequate calcium intake is important for the prevention of hypertension and preeclampsia and for bone health maintenance, and it has also been associated with the reduction of renal stones, cholesterol values, and colorectal cancer.^25,29,40-44^ It has been reported that calcium can interfere with iron absorption in the short term; however, prolonged calcium supplementation has proven to have no effect on iron status over time.^45-50^ Concerns on the potential increase in atherosclerotic vascular disease in women receiving calcium supplements, allayed by a systematic review and meta-analysis including 18 RCTs involving 63 564 elderly women participants, concluded that calcium supplementation with or without vitamin D has no effect on coronary heart disease or all-cause mortality risk.^51-54^

With the current high prevalence of overweight and obesity among women of childbearing age and low calcium intake in numerous low- and middle-income countries, calcium supplementation might aid weight management before and during pregnancy and assist women to reach their pregnancy with healthier body weights.^55,56^

### Strengths

This is a RCT with standardized procedures used in all sites. Women in this study had close follow-up, and weight and height were measured by the same research team before and during pregnancy.

### Limitations

An important limitation of this analysis is the small sample size. Our subanalysis was not powered to detect weight reduction differences smaller than 1.6 kg that are also clinically relevant at a population level. It is important to note that the findings of this study are only applicable to women with a risk of preeclampsia and not generalizable to all pregnant women. It has been shown that obesity, hypertension, and endothelial dysfunction are risk factors for preeclampsia. In this way, women in the CAP trial might have had an increased occurrence of these diseases compared to the general population and therefore different physiological changes.^57,58^ In addition, we only had the admission measurement of body weight before pregnancy for each participant, and thus, we used body weight measured at 8 weeks as a proxy of prepregnancy body weight. Finally, we only measured diet at 20 weeks’ gestation when all women had finished the trial intervention, which limited the analysis we can perform with dietary information.

## Conclusion

Even though the clinical relevance of a small weight reduction at individual level has been questioned, at a population level, it could help to prevent the observed obesity global trends.^23^ We found that a low dose of calcium supplementation of 500 mg of elemental calcium per day had no statistically significant effect on body weight in women preconceptionally and during early pregnancy. However, the smaller increase in body weight we found in women supplemented with calcium is consistent with previous human studies and also is also biologically plausible based upon prior mechanistic studies. Data from this study will contribute to the overall body of evidence on this topic, including systematic reviews of randomized trials.

## Supplemental Material

Supplemental Material, 2019-10-04_STable_1 - The Effect of Calcium Supplementation on Body Weight Before and During Pregnancy in Women Enrolled in the WHO Calcium and Preeclampsia Trial Supplemental Material, 2019-10-04_STable_1 for The Effect of Calcium Supplementation on Body Weight Before and During Pregnancy in Women Enrolled in the WHO Calcium and Preeclampsia Trial by Gabriela Cormick, Ana Pilar Betrán, Janetta Harbron, Armando Seuc, Cintia White, James M. Roberts, Jose M. Belizán, G. Justus Hofmeyr and on behalf of the CAP Study Group in Food and Nutrition Bulletin

Supplemental Material, 2019-10-04_STable_2 - The Effect of Calcium Supplementation on Body Weight Before and During Pregnancy in Women Enrolled in the WHO Calcium and Preeclampsia Trial Supplemental Material, 2019-10-04_STable_2 for The Effect of Calcium Supplementation on Body Weight Before and During Pregnancy in Women Enrolled in the WHO Calcium and Preeclampsia Trial by Gabriela Cormick, Ana Pilar Betrán, Janetta Harbron, Armando Seuc, Cintia White, James M. Roberts, Jose M. Belizán, G. Justus Hofmeyr and on behalf of the CAP Study Group in Food and Nutrition Bulletin

Supplemental Material, 2019-10-04_STable_3 - The Effect of Calcium Supplementation on Body Weight Before and During Pregnancy in Women Enrolled in the WHO Calcium and Preeclampsia Trial Supplemental Material, 2019-10-04_STable_3 for The Effect of Calcium Supplementation on Body Weight Before and During Pregnancy in Women Enrolled in the WHO Calcium and Preeclampsia Trial by Gabriela Cormick, Ana Pilar Betrán, Janetta Harbron, Armando Seuc, Cintia White, James M. Roberts, Jose M. Belizán, G. Justus Hofmeyr and on behalf of the CAP Study Group in Food and Nutrition Bulletin

## Appendix A

### Calcium And Preeclampsia Study Group

Argentina: Fernando Althabe, José M Belizán, Eduardo Bergel, Alvaro Ciganda, Gabriela Cormick. Canada: Diane Sawchuck, Marianne Vidler. South Africa: Saadiqa Allie, John Anthony, Karlyn Frank, Annmarie de Greeff, Sue Fawcus, David Hall, Justus Hofmeyr, Mvuseleli Kovane, Patience Kovane, Theresa Lawrie, Simpiwe Mose, Nolundi Mshweshwe, Velisa Mqikela, Pamela Njikelana, Natalia Novikova, Adegboyega Oyebajo, Catherine Parker, Angel Phuti, Mandisa Singata-Madliki, Erika van Papendorp, Xoliswa Williams. Switzerland: Ana Pilar Betrán, Tina Dannemann, Armando Seuc. United Kingdom: Laura Magee, Peter von Dadelszen. USA: France Donnay, Sharla Drebit, Jim Roberts. Zimbabwe: Bothwell Guzha, Emilia Makaza, Sarah Manyame, Stephen Munjanja, Eunice Tahuringana.
