# Supplementary material for: The Effect of Calcium Supplementation on Body Weight Before and During Pregnancy in Women Enrolled in the WHO Calcium and Preeclampsia Trial
Source: Food Nutr Bull. 2020 Nov 17;41(3):332–42. doi: 10.1177/0379572120944671 (PMC11951462; doi:10.1177/0379572120944671)
Supplement: Supplemental Material, 2019-10-04_STable_1 - The Effect of Calcium Supplementation on Body Weight Before and During Pregnancy in Women Enrolled in the WHO Calcium and Preeclampsia Trial [file 2019-10-04_STable_1.pdf]

Table S1: Participants compliance of 80% or more of the study supplements

	PLACEBO			CALCIUM			
Compliance (>80% pills taken)	N	n	%	N	n	%	p value*
From last PPV[§] up to before 8 weeks	227	131	57.7	230	122	53	0.363
From last PPV up to 20 weeks	198	117	59.1	198	118	59.6	1
From last PPV up to 32 weeks	139	72	51.8	142	79	55.6	0.629

*Differences were tested using a chi^2 test and a p value of 0.05

[§]PPV = Pre-pregnancy visit
