# Supplementary material for: The Effect of Calcium Supplementation on Body Weight Before and During Pregnancy in Women Enrolled in the WHO Calcium and Preeclampsia Trial
Source: Food Nutr Bull. 2020 Nov 17;41(3):332–42. doi: 10.1177/0379572120944671 (PMC11951462; doi:10.1177/0379572120944671)
Supplement: Supplemental Material, 2019-10-04_STable_2 - The Effect of Calcium Supplementation on Body Weight Before and During Pregnancy in Women Enrolled in the WHO Calcium and Preeclampsia Trial [file 2019-10-04_STable_2.pdf]

Table S2: Weight change between admission and 8, 20 and 32 weeks´ gestation by baseline Body Mass Index (BMI) in those that had complied with 80% or more of supplements intake from admission to each corresponding visit

	PLACEBO		CALCIUM			
	n	Mean difference (SD)	n	Mean difference (SD)	Mean difference (95% CI)	p value*
**Weight change at 8 weeks gestation**	131	1.1 (4.4)	122	1.4 (5.4)	0.34 (-0.87 to 1.55)	0.578
BMI[§] at ADM[⌘] < 25 kg/m2	44	1.1 (4.4)	35	1.5 (4.8)	0.42 (-1.64 to 2.48)	0.688
25<=BMI at ADM<30 kg/m2	42	1.7 (4.1)	39	1.1 (3.2)	-0.59 (-2.22 to 1.04)	0.476
BMI at ADM>=30 kg/m2	45	0.5 (4.7)	48	1.6 (7.0)	1.11 (-1.36 to 3.59)	0.374
**Weight change at 20 weeks gestation**	117	3.4 (5.7)	118	3.6 (5.6)	0.12 (-1.32 to 1.57)	0.867
BMI at ADM < 25 kg/m2	35	4.5 (6.4)	39	4.1 (4.3)	-0.36 (-2.88 to 2.15)	0.774
25<=BMI at ADM<30 kg/m2	41	3.4 (5.3)	29	3.2 (4.2)	-0.24 (-2.61 to 2.13)	0.839
BMI at ADM>=30 kg/m2	41	2.5 (5.1)	50	3.3 (7.1)	0.79 (-1.85 to 3.42)	0.554
**Weight change at 32 weeks gestation**	72	8.4 (7.8)	79	7.7 (5.6)	-0.64 (-2.81 to 1.52)	0.558
BMI at ADM < 25 kg/m2	22	6.6 (5.1)	24	9.2 (3.8)	2.53 (-0.16 to 5.22)	0.065
25<=BMI at ADM<30 kg/m2	27	9.0 (4.9)	20	8.8 (4.3)	-0.16 (-2.91 to2.59)	0.906
BMI at ADM>=30 kg/m2	23	9.4 (11.7)	35	6.2 (6.8)	-3.23 (-8.12 to 1.65)	0.190

* Differences were tested using a t-test and a p value of 0.05

[§] BMI= Body Mass Index
[⌘] ADM= Admission
