# Supplementary material for: The Effect of Calcium Supplementation on Body Weight Before and During Pregnancy in Women Enrolled in the WHO Calcium and Preeclampsia Trial
Source: Food Nutr Bull. 2020 Nov 17;41(3):332–42. doi: 10.1177/0379572120944671 (PMC11951462; doi:10.1177/0379572120944671)
Supplement: Supplemental Material, 2019-10-04_STable_3 - The Effect of Calcium Supplementation on Body Weight Before and During Pregnancy in Women Enrolled in the WHO Calcium and Preeclampsia Trial [file 2019-10-04_STable_3.pdf]

Table S3: Weight change in kilograms from admission to 8 weeks' gestation by duration of supplementation

	PLACEBO		CALCIUM		
	n	Mean difference (SD)	n	Mean difference (SD)	p value*
**Weight change at 8 weeks´ gestation**					
Time in study < 2 months	6	1.2 (2.6)	5	-0.2 (2.7)	0.418
Time in study 2 to 6 months	81	0.6 (3.9)	77	0.3 (4.2)	0.635
Time in study 6 to 12 months	55	1.3 (3.7)	72	1.3 (5.7)	0.957
Time in study >=12	85	2.5 (8.6)	76	1.7 (6.6)	0.511

* Differences were tested using a t-test and a p value of 0.05
